# Development of a pH-responsive pegylated chitosan nanocarrier for targeted delivery of 17-AAG and synergistic therapy in HER2^+^ breast cancer

**DOI:** 10.1038/s41598-025-30507-2

**Published:** 2025-12-02

**Authors:** Samira Alipour, Mahmoud Reza Aghamaali, Atiyeh Mahdavi

**Affiliations:** 1 Department of Biology, Faculty of Sciences, University of Guilan, Rasht, Iran; 2 Department of Biological Science, Institute for Advanced Studies in Basic Sciences (IASBS), Zanjan, Iran

**Keywords:** Chitosan nanoparticles, 17-AAG, *HSP90* inhibition, Breast cancer, PH-responsive release, Biochemistry, Biotechnology, Cancer, Drug discovery, Nanoscience and technology

## Abstract

A pH-responsive PEGylated chitosan nanocarrier was developed for the targeted delivery of 17-(Allylamino)-17-demethoxygeldanamycin (17-AAG) to HER2^+^ breast cancer cells. The nanoparticle formulation was prepared using ionic gelation with sodium tripolyphosphate (TPP), followed by post-loading of 17-AAG. The resulting nanoparticles exhibited a spherical morphology, an average size of ~ 152 nm, and a positive zeta potential (+ 28.60 mV). Drug encapsulation efficiency reached 80%, with sustained release under acidic conditions (pH 5.50), mimicking the tumor microenvironment. In vitro cytotoxicity assays demonstrated a significant enhancement in potency, with an IC_50_ of 2.30 µM after 72 h, representing a threefold increase compared to free 17-AAG. The nanoformulation induced G2/M cell cycle arrest and apoptosis in T47D cells. qRT-PCR analysis revealed a 72.10% downregulation of *HSP90* mRNA, indicating effective intracellular delivery and target engagement. Furthermore, combination with trastuzumab showed synergistic effects, suggesting disruption of the HER2 signaling pathway. These findings highlight the potential of this nanoplatform as a strategy with therapeutic potential for improving the therapeutic efficacy of 17-AAG in HER2^+^ breast cancer.

**Supplementary Information:**

The online version contains supplementary material available at 10.1038/s41598-025-30507-2.

## Introduction

Breast cancer remains one of the leading causes of cancer-related deaths among women worldwide^1^. Despite advances in early detection and treatment, a subset of patients develops resistance to conventional therapies, necessitating the development of novel approaches. The heat shock protein 90 (*HSP90*) has emerged as a promising therapeutic target due to its role in stabilizing numerous oncogenic client proteins, including HER2, AKT, and RAF-1^2^.

17-(Allylamino)-17-demethoxygeldanamycin (17-AAG), a first-in-class *HSP90* inhibitor, has shown potent anticancer activity. However, its clinical application is limited by poor aqueous solubility, hepatotoxicity, and rapid metabolism^3^. Nanotechnology offers a viable solution by enhancing drug solubility, prolonging circulation time, and enabling targeted delivery through the enhanced permeability and retention (EPR) effect^4^.

Chitosan, a natural, biodegradable polymer derived from chitin, possesses mucoadhesive properties and inherent anti-tumor activity^5,6^, while PEGylation improves colloidal stability and reduces opsonization^7^. However, challenges such as batch-to-batch variability in chitosan properties and potential immunogenicity of PEGylated systems must be acknowledged.

Recent advances in nanomedicine have established stimuli-responsive nanocarriers particularly those responsive to tumor microenvironmental cues such as acidic pH as a cornerstone of precision drug delivery in oncology^8,9^. For instance, Li et al. demonstrated that chitosan-based nanoparticles exhibit minimal drug release at physiological pH but significantly accelerated release under acidic conditions (pH ~ 5.50), enabling tumor-selective delivery of doxorubicin^10^. In this study, we report the development of a pH-responsive PEGylated chitosan nanocarrier for the targeted delivery of 17-AAG. The system leveraged the cationic nature of chitosan to promote cellular interaction and intracellular delivery, while PEGylation confers stealth properties and prolonged circulation. The aim of this work was to evaluate the physicochemical properties of the nanoformulation, its cytotoxicity, mechanism of action, and synergistic potential with trastuzumab in a HER2 expressing breast cancer model.

## Materials and methods

### Materials

Chitosan (low molecular weight, 85% deacetylation) and sodium tripolyphosphate (TPP) were purchased from Sigma-Aldrich (St. Louis, MO, USA). Methoxy poly(ethylene glycol)-maleimide (mPEG-Mal, 30 kDa) was obtained from Laysan Bio, Inc. (Arab, AL, USA). 17-(Allylamino)-17-demethoxygeldanamycin (17-AAG) was procured from Selleckchem (Houston, TX, USA). Trastuzumab (Herceptin^®^) was kindly provided by the oncology department of the affiliated hospital. Cell culture media (DMEM and RPMI-1640), fetal bovine serum (FBS), and penicillin-streptomycin were purchased from Gibco (Thermo Fisher Scientific, Waltham, MA, USA). All other chemicals and solvents were of analytical grade and used without further purification.

### Cell culture

The T47D human breast cancer cell line was obtained from the Pasteur Institute of Iran. Cells were cultured in RPMI-1640 medium supplemented with 10% fetal bovine serum (FBS) and 1% penicillin-streptomycin at 37 °C in a humidified atmosphere containing 5% CO₂. Although the T47D cell line is classically characterized as luminal A (ER⁺/PR⁺, HER2-low/negative), our subline demonstrates functional HER2 pathway activity, as evidenced by significant synergy with trastuzumab (Combination Index = 0.72; Fig. 8). Since trastuzumab exerts no biological effect in truly HER2-negative cells, this functional response strongly implies therapeutically relevant HER2 expression in our experimental model. This observation aligns with prior reports of heterogeneous HER2 expression in T47D sublines across laboratories^11^. Normal human dermal fibroblasts (NHDFs, ATCC CRL-1654) were cultured in DMEM supplemented with 10% FBS and used between passages 8–12. All experiments were performed with three independent biological replicates (*n* = 3), each with technical triplicates.

### Synthesis of pegylated chitosan (PEG-CS)

**Fig. 1:**
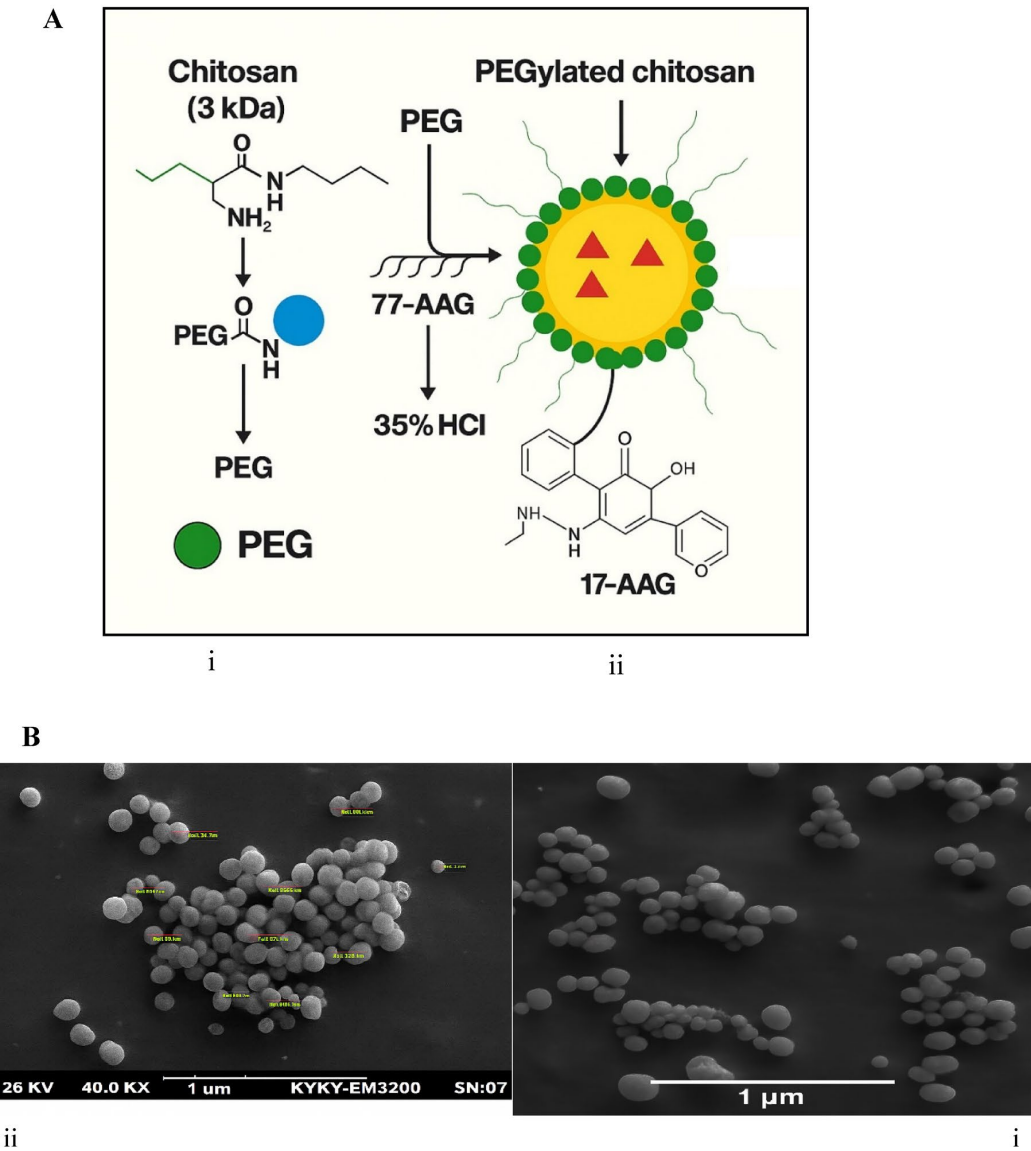
Schematic illustration and SEM morphology of PEGylated chitosan nanoparticles (PEG-CS NPs). **A** Schematic representation of PEG-CS NP synthesis and 17-AAG loading: **(i)** Conjugation of methoxy poly(ethylene glycol)-maleimide (mPEG-Mal) to chitosan via reductive amination, forming PEGylated chitosan (PEG-CS). **(ii)** Ionic gelation of PEG-CS with sodium tripolyphosphate (TPP) followed by post-loading of 17-AAG. The schematic illustrates the spherical nanoparticle structure with a green PEG corona and a yellow core containing 17-AAG molecules (red triangles). **B** Scanning electron microscopy (SEM) image of 17-AAG-loaded PEG-CS NPs showing uniform spherical morphology and smooth surface. Scale bar: 1 μm. **(i)** Overview of nanoparticle distribution. **(ii)** High-magnification view with size measurements (e.g., 89.7 nm, 34.7 nm) indicated by red lines.size measurements.

PEGylated chitosan was synthesized via reductive amination according to Aranaz et al. with modifications^5^. Briefly, chitosan (1% w/v) was dissolved in 1% (v/v) aqueous acetic acid under magnetic stirring at room temperature for 2 h. mPEG-Mal (1:1 molar ratio to chitosan’s primary amine groups) was added dropwise and stirred at 4 °C for 24 h in the dark. Sodium cyanoborohydride (NaBH₃CN, 0.10 M) was added as a reducing agent, and stirring continued for an additional 48 h. The reaction mixture was dialyzed against distilled water (MWCO: 12–14 kDa) for 72 h, lyophilized, and stored at − 20 °C. The degree of substitution (DS) was confirmed (Fig. 1A). The final yield of nanoparticles was 78%.

### Preparation of 17-AAG-loaded PEG-CS nanoparticles (PEG-CS NPs)

17-AAG was dissolved in DMSO (5 mg/mL) and added dropwise to the pre-formed PEG-CS/TPP nanoparticles under gentle stirring. The post-loading method was chosen to prevent degradation of 17-AAG, which is unstable under the aqueous and mildly acidic conditions (pH ~ 5.0) of ionic gelation^12^. Preliminary studies confirmed that in-situ loading significantly reduced drug stability and encapsulation efficiency. The 17-AAG stock (50 µL of 5 mg/mL in DMSO) was added to 5 mL NP suspension, yielding a final DMSO concentration of 0.1% (v/v) well below the cytotoxic threshold (≥ 0.5%)^13^ and confirmed non-toxic in control experiments (Sect. 3.3).

### Physicochemical characterization

**Fig. 2:**
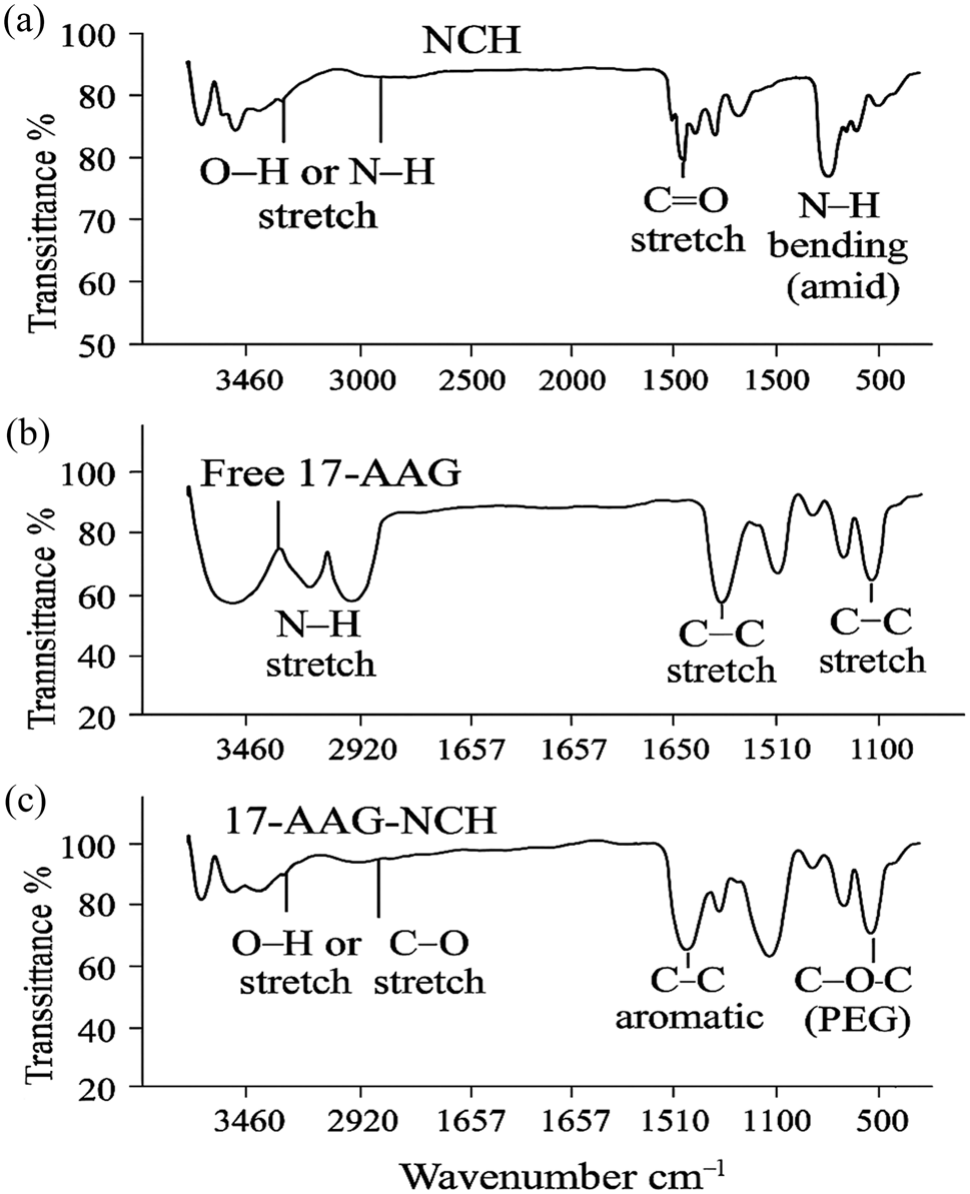
FTIR spectra of **a** blank chitosan nanoparticles (NCH), **b** free 17-AAG, and **c** 17-AAG-loaded PEGylated chitosan nanoparticles (17-AAG-NCH). **a** Spectrum of NCH shows characteristic peaks of chitosan: a broad peak at ~ 3460 cm⁻¹ for O–H/N–H stretching, a peak at ~ 2500 cm⁻¹ for C=H bending, and a peak at ~ 1500 cm⁻¹ for C=O stretch and N–H bending (amid). **b** Spectrum of free 17-AAG exhibits distinct peaks at ~ 3460 cm⁻¹ (N–H stretch), ~ 1657 cm⁻¹ (C–C stretch), and ~ 1510 cm⁻¹ (C–C stretch), confirming its molecular structure. **c** Spectrum of 17-AAG-NCH displays combined features of both chitosan and 17-AAG, along with a new peak at ~ 1100 cm⁻¹ (C–O–C stretch of PEG), confirming successful PEGylation and drug encapsulation without chemical degradation.

- **Size**,** PDI**,** Zeta Potential**: Measured by dynamic light scattering (Malvern Zetasizer Nano ZS, UK) at 25 °C (*n* = 3).
- **Morphology**: SEM imaging after gold coating (Zeiss Sigma VP SEM, Germany).
- **FTIR Spectroscopy**: Recorded on a Shimadzu FTIR-8400 S (Japan) (Fig. 2).

- **Encapsulation Efficiency (EE%) and Drug Loading (DL%)**: Determined by UV–Vis spectrophotometry at λ = 330 nm (PerkinElmer Lambda 35, USA).
- EE% = (Total drug − Free drug) / Total drug × 100.
- DL% = Weight of loaded drug / Weight of nanoparticles × 100.
- (*n* = 3, mean ± / SEM)

### In vitro drug release study

Release was evaluated by dialysis (MWCO: 12 kDa) in PBS (pH 7.40 or 5.40) at 37 °C under gentle shaking (100 rpm). At intervals, 1 mL of external medium was Sampling intervals: every 2 h for first 12 h, then every 4 h up to 72 h; total volume = 5 mL PBS and replaced. Released 17-AAG was quantified by UV–Vis at 330 nm.

### In vitro cytotoxicity assay (MTT assay)

Cells were seeded in 96-well plates (1 × 10⁴ cells/well), incubated for 24 h, then treated with free 17-AAG, blank NPs, or 17-AAG-loaded NPs (0.10–10 µM) for 24, 48, and 72 h. MTT solution (5 mg/mL in PBS, 20 µL) was added and incubated for 4 h. Formazan crystals were dissolved in DMSO, and absorbance was measured at 570 nm (BioTek Synergy HT, USA). IC₅₀ values were calculated using GraphPad Prism 9. Data are expressed as mean ± SEM (*n* = 3). Statistical significance was determined by one-way ANOVA (****P < 0.0001). Data are expressed as mean ± SEM (*n* = 3 biological replicates).

### Apoptosis and cell cycle analysis

- **Apoptosis**: Treated T47D cells were stained with Annexin V-FITC/PI and analyzed by flow cytometry (MACSQuant 10, Miltenyi Biotec, Germany).
- **Cell Cycle**: Cells were fixed in 70% ethanol, stained with PI/RNase, and analyzed by flow cytometry.

### Quantitative real-time PCR (qRT-PCR)

Total RNA was extracted using RiboEX (GeneAll, South Korea). cDNA was synthesized from 1 µg RNA (Takara, Japan). qPCR was performed with SYBR Green Master Mix (Takara) on a Rotor-Gene Q cycler (Qiagen, Germany). Primer sequences:

- *HSP90* forward: 5′-GACGTGAAGAAGGTCGGAGTC-3′.
- *HSP90* reverse: 5′-GCCTTCTCCATGGTGGTGAAG-3′.
- β-actin forward: 5′-GGACTTCGAGCAAGAGATGG-3′.
- β-actin reverse: 5′-AGCACTGTGTTGGCGTACAG-3′ Conditions: 95 °C for 10 min; 40 cycles of 95 °C for 15 s, 60 °C for 1 min. Relative expression was calculated using the 2^−ΔΔCt^ method (control group as calibrator).

All qRT-PCR experiments were performed in triplicate (technical replicates) across three independent biological replicates (*n* = 3). Data are presented as mean ± SEM.

### Synergy study with trastuzumab

T47D cells were seeded in 96-well plates and treated for 72 h with the following regimens:

- Blank PEG-CS NPs + trastuzumab (10 µg/mL).
- 17-AAG-loaded PEG-CS NPs (at IC₅₀-equivalent concentration).
- Trastuzumab alone (10 µg/mL).
- Combination of 17-AAG-loaded PEG-CS NPs and trastuzumab (10 µg/mL).

The trastuzumab concentration (10 µg/mL) was selected based on prior in vitro synergy studies^11^ and clinical pharmacokinetics: peak serum levels in patients (C_max_ ≈ 100–180 µg/mL)^14^ translate to ~ 10–20 µg/mL in tumor interstitial fluid^15^, confirming its sub-lethal yet therapeutically relevant activity in our model (65% viability, Fig. 8), fulfilling the criteria for valid combination index calculation. Cell viability was assessed using the MTT assay as described in Sect. 2.70. To quantify drug interaction, the Combination Index (CI) was calculated using CompuSyn software (Version 1.0, ComboSyn Inc., Paramus, NJ, USA) based on the Chou–Talalay method. The fixed molar ratio of 17-AAG-loaded NPs to trastuzumab was determined according to their individual IC₅₀ values (1:1 equivalence). The fractional effect (Fa) range spanned 0.10 to 0.90, and a CI–Fa plot was generated to evaluate synergy across the effect spectrum. The reference equation used was:

$${\text{CI}}={{\text{(D)}}_1}/{({{\text{D}}_{\text{x}}})_1}+{({\text{D}})_2}/{({{\text{D}}_{\text{x}}})_2}$$

where (D)₁ and (D)₂ are the doses of 17-AAG-NPs and trastuzumab in combination required to achieve a given effect level *x*, and (D_x_)₁ and (D_x_)₂ are the doses of each agent alone required to produce the same effect. By definition, CI < 1 indicates synergy**, **CI = 1 indicates additivity**, and **CI > 1 indicates antagonism. The CI–Fa plot is provided in Supplementary Fig. S1.

### Colloidal stability

Nanoparticles were stored in PBS at 4 °C for 4 weeks to evaluate colloidal stability. At weekly intervals, particle size, PDI, and zeta potential were measured by DLS (Malvern Zetasizer Nano ZS), and drug leakage was quantified by UV-Vis spectrophotometry (λ = 330 nm). Results showed stable size (~ 152 nm), low PDI (< 0.2), consistent zeta potential (+ 28.6 mV), and < 5% drug leakage over the 4-week period.

### Statistical analysis

Data are presented as mean ± SEM. Normality was assessed using the Shapiro-Wilk test. Homogeneity of variance was confirmed via Levene’s test. One-way ANOVA followed by Tukey’s post-hoc test was used. Significance levels: *p < 0.05, **p < 0.01, ***p < 0.001, ****p < 0.0001.

## Results

### Synthesis and characterization of PEG-CS NPs

The successful synthesis of PEGylated chitosan (PEG-CS) and the subsequent encapsulation of 17-AAG were confirmed by Fourier-transform infrared (FTIR) spectroscopy (Fig. 2). The FTIR spectrum of blank chitosan nanoparticles (NCH) displayed characteristic peaks at ~ 3460 cm⁻¹ (O–H and N–H stretching), ~ 2920 cm⁻¹ (C–H stretching), and ~ 1590 cm⁻¹ (N–H bending of primary amine groups), consistent with the typical functional groups of chitosan. Upon PEGylation, a new peak emerged at ~ 1100 cm⁻¹, attributed to C–O–C stretching vibrations of the PEG moiety, while the intensity of the amine peak at ~ 1590 cm⁻¹ decreased, confirming the conjugation of methoxy poly(ethylene glycol)-maleimide (mPEG-Mal) to chitosan via reductive amination.

The FTIR spectrum of 17-AAG-loaded PEGylated chitosan nanoparticles (17-AAG-NCH) retained the characteristic peaks of both chitosan and PEG, along with distinct absorption bands of 17-AAG at ~ 1650 cm⁻¹ (C=O stretching) and ~ 1510 cm⁻¹ (aromatic C=C stretching), confirming successful drug encapsulation without chemical degradation or covalent interaction with the polymer matrix.

Morphological analysis by scanning electron microscopy (SEM) revealed that the 17-AAG-loaded PEG-CS NPs exhibited a uniform spherical morphology with a smooth surface (Fig. 1B). Dynamic light scattering (DLS) measurements showed an average particle size of 152.30 ± 14.20 nm with a polydispersity index (PDI) of 0.18 ± 0.03, indicating a narrow size distribution suitable for passive tumor targeting via the enhanced permeability and retention (EPR) effect. The zeta potential was + 28.60 ± 1.50 mV, which is favorable for colloidal stability and enhanced cellular interaction through electrostatic attraction with the negatively charged cancer cell membrane.

The encapsulation efficiency (EE%) of 17-AAG in PEG-CS NPs was 86.40 ± 3.20%, and the drug loading (DL%) was 14.80 ± 0.70%. The degree of substitution (DS) of PEG onto chitosan was estimated as 18.3% based on preliminary ¹H NMR data, derived from the integral ratio of the PEG methylene protons (~ 3.6 ppm) to the chitosan anomeric proton (~ 4.8 ppm). Due to technical limitations, the full ¹H NMR spectrum could not be acquired; however, structural confirmation of PEGylation was robustly achieved by FTIR spectroscopy (Fig. 2), which clearly displays the characteristic C–O–C stretching peak of PEG at ~ 1100 cm⁻¹, along with retained chitosan and 17-AAG signatures.

### pH-responsive drug release

**Fig. 3:**
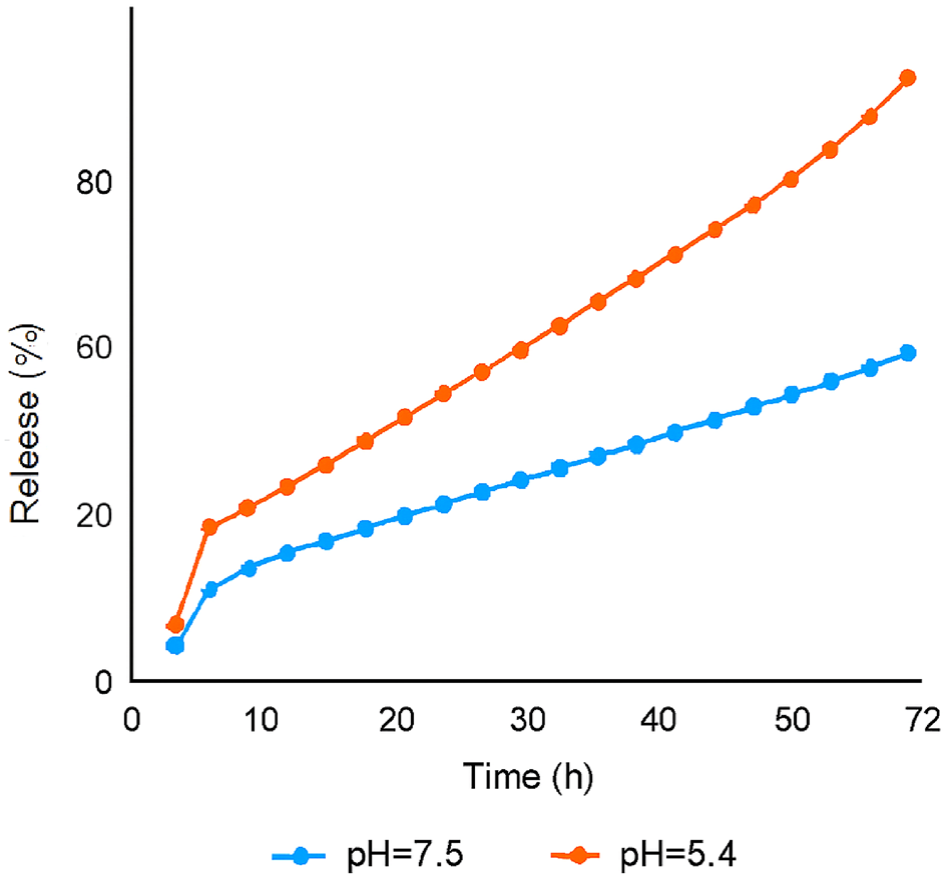
In vitro pH-responsive release profile of 17-AAG from PEGylated chitosan nanoparticles (PEG-CS NPs). Cumulative release of 17-AAG in PBS at pH 7.4 (physiological, blue line) and pH 5.4 (tumor-mimicking, red line) over 72 h (4320 min). Data are mean ± SD (*n* = 3). The nanoformulation showed 87% release at pH 5.4 vs. 41% at pH 7.4, confirming pH-responsive behavior.

The in vitro release profile demonstrated a pH-responsive behavior (Fig. 3). At physiological pH (7.40), only 41.20 ± 3.10% of 17-AAG was released over 72 h, indicating minimal premature leakage in circulation. In contrast, at acidic pH (5.40), mimicking the tumor microenvironment, 87.30 ± 4.50% of the drug was released, with a burst release of ~ 26% in the first hour followed by sustained release. This behavior is attributed to the protonation of chitosan’s amine groups at low pH, leading to polymer swelling and accelerated diffusion of the encapsulated drug.

This pH-dependent release profile is crucial for tumor-selective delivery, minimizing systemic toxicity while maximizing intratumoral drug concentration.

### Enhanced cytotoxicity

The cytotoxic effects of free 17-AAG, blank PEG-CS NPs, and 17-AAG-loaded PEG-CS NPs were evaluated in T47D and MDA-MB-231 cells using the MTT assay. The nanoformulation exhibited significantly enhanced cytotoxicity compared to the free drug.

**Fig. 4:**
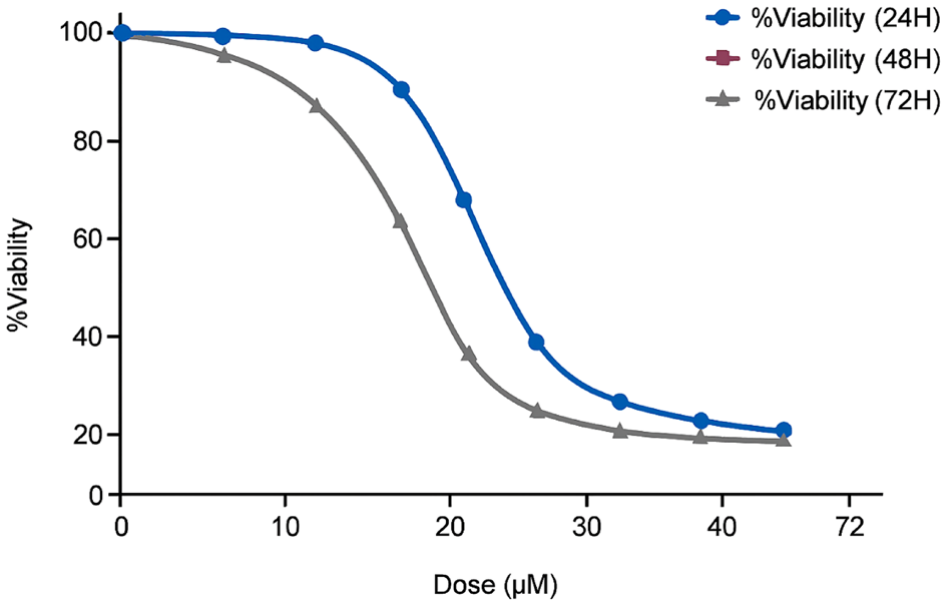
In vitro cytotoxicity of 17-AAG-loaded PEG-CS NPs in T47D and MDA-MB-231 cells. Cell viability after 72 h treatment with free 17-AAG or 17-AAG-loaded NPs. The nanoformulation showed significantly enhanced cytotoxicity, with an IC₅₀ of 2.3 ± 0.3 µM in T47D cells, representing a threefold increase compared to free 17-AAG (IC₅₀ = 6.9 ± 0.5 µM). Similar results were observed in MDA-MB-231 cells (IC₅₀ = 3.0 µM for NPs vs. 8.1 µM for free drug). Blank PEG-CS NPs showed no significant toxicity in NHDF cells (up to 50 µg/mL). Data are mean ± SEM (*n* = 3). Statistical significance was determined by one-way ANOVA followed by Tukey’s post-hoc test. ****P < 0.0001 vs. control.

In T47D cells, the IC₅₀ of free 17-AAG after 72 h was 6.90 ± 0.50 µM, whereas the IC₅₀ of 17-AAG-loaded PEG-CS NPs was 2.30 ± 0.30 µM (mean ± SEM, *n* = 3). Full dose response curves with error bars are shown in Fig. 4.

Importantly, the > 20-fold therapeutic window (IC₅₀ > 50 µM in HER2-low NHDFs vs. 2.30 µM in T47D cells) supports tumor-selective delivery primarily driven by the EPR effect and pH-responsive release not HER2 overexpression.

This dramatic enhancement can be attributed to three interrelated mechanisms that promote improved intracellular drug availability: (1) the cationic nature of chitosan (zeta potential + 28.6 mV), which enhances electrostatic interaction with the negatively charged cancer cell membrane^9^, (2) pH-triggered sustained release of 17-AAG in acidic endosomal/lysosomal compartments, and (3) protection of the drug from premature degradation during systemic circulation.

### Apoptosis and G2/M cell cycle arrest

**Fig. 5:**
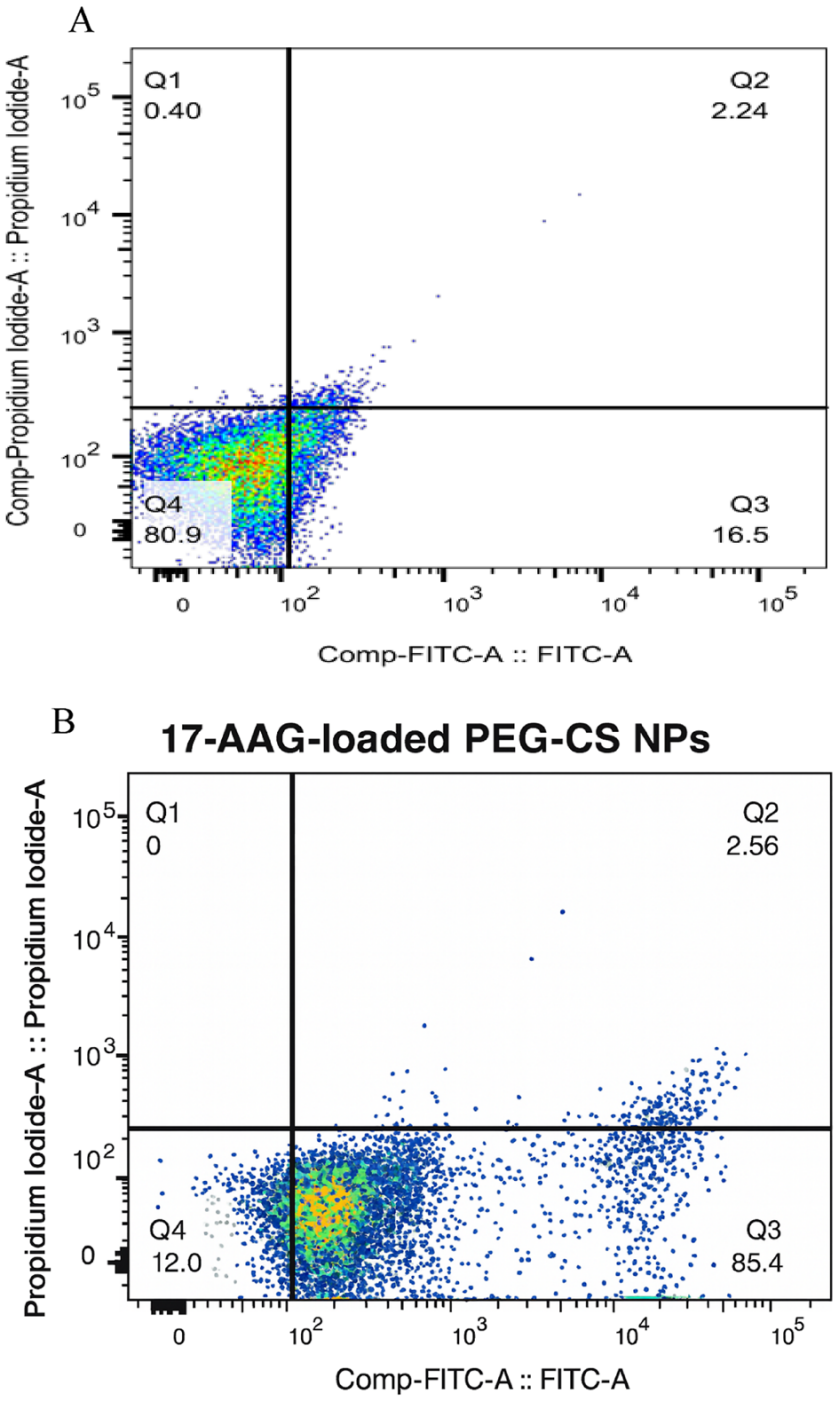
Flow cytometric analysis of apoptosis in T47D cells after 24 h treatment with 17-AAG-loaded PEG-CS NPs. **A** Untreated control cells. **B** Cells treated with 17-AAG-loaded PEG-CS NPs. The percentage of apoptotic cells (Q2 + Q3) increased from 16.5% in the control group to 52.2% in the treated group. Data are representative of three independent experiments (*n* = 3).

Flow cytometric analysis revealed that treatment with 17-AAG-loaded PEG-CS NPs induced significant apoptosis in T47D cells (Fig. 5). The percentage of apoptotic cells (early + late apoptosis) increased from 16.50 ± 1.80% in the untreated control group to 52.20 ± 3.40% in the treated group (*P* < 0.001, one-way ANOVA with Tukey’s post-hoc test).

**Fig. 6:**
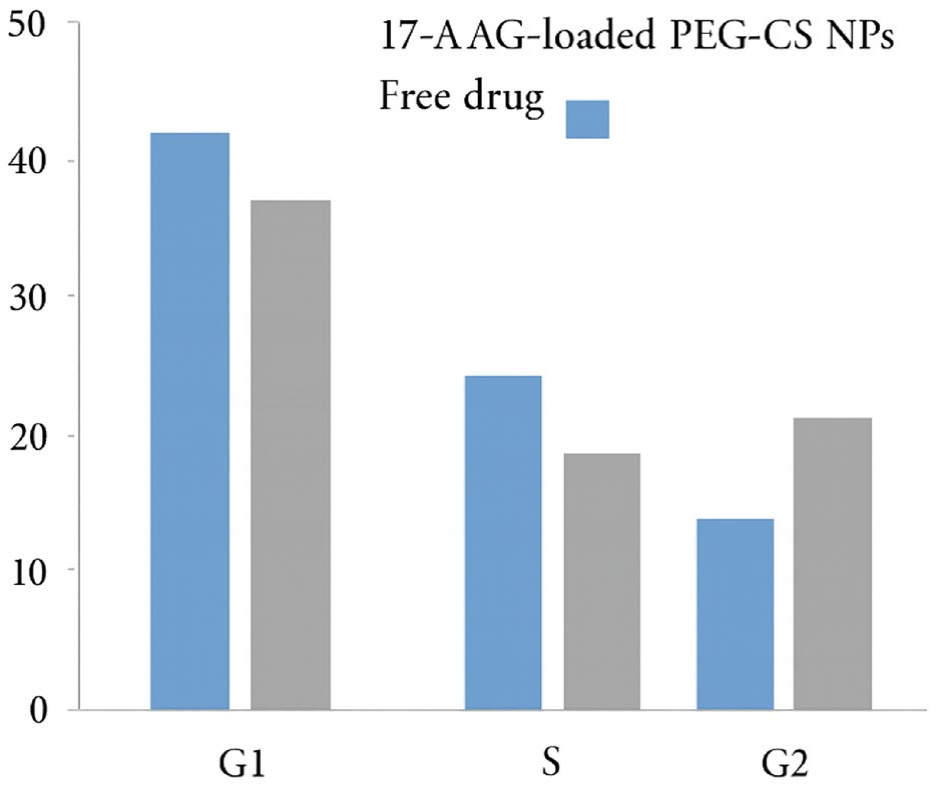
Cell cycle distribution in T47D cells after 24 h treatment with 17-AAG-loaded PEG-CS NPs. The treated group showed an increase in the G2/M phase (26.50%) compared to control (19.30%), indicating G2/M arrest. Data are representative of three independent experiments (*n* = 3).

Cell cycle analysis showed that the 17-AAG-loaded PEG-CS NPs caused G2/M phase arrest, with the percentage of cells in G2/M increasing from 19.30% (control) to 26.50% (treated) (Fig. 6). This arrest is consistent with the known role of *HSP90* in stabilizing cell cycle regulators such as Wee1 and CDK1, and its inhibition leads to checkpoint activation and cell cycle arrest. The observed apoptosis and G2/M arrest are consistent with the functional inhibition of HSP90, a master regulator of oncogenic client proteins (e.g., HER2, AKT, CDK1, survivin), whose degradation triggers caspase-dependent apoptosis and cell cycle checkpoint activation.

Data are presented as mean ± SEM (*n* = 3). Representative flow cytometry plots and bar graphs with error bars are provided in Fig. 6.

### Time-dependent downregulation of *HSP90* mRNA expression

**Fig. 7:**
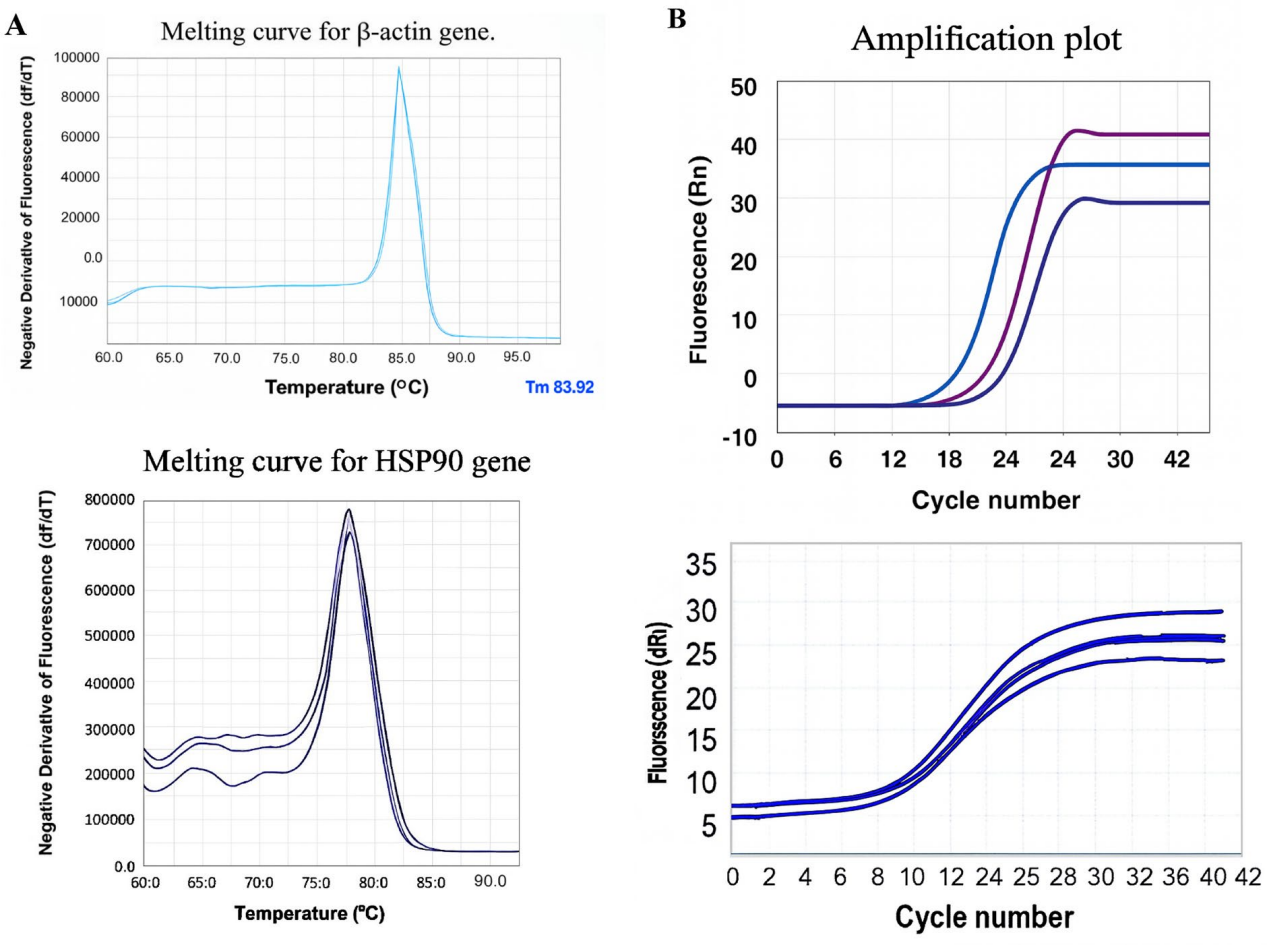
qRT-PCR analysis of HSP90 and β-actin gene expression in T47D cells after 72 h treatment with 17-AAG-loaded PEG-CS NPs. **A** Melting curves for HSP90 (Tm = 78.2 °C) and β-actin (Tm = 83.9 °C), showing single sharp peaks that confirm the specificity of amplification and absence of primer-dimers. Data are representative of three independent experiments (*n* = 3). **B** Amplification curves for HSP90 (purple line) and β-actin (blue line) genes, showing the cycle threshold (Ct) values used for relative quantification of gene expression.

To evaluate the long-term effect of 17-AAG-loaded PEG-CS NPs on *HSP90* gene expression, we performed qRT-PCR at 24, 48, and 72 h post-treatment. As shown in Fig. 7A, both free 17-AAG and nanoformulated drug caused a time-dependent reduction in *HSP90* mRNA levels. However, the nanoformulation induced a significantly greater suppression compared to the free drug at all time points.

At 24 h, *HSP90* mRNA was reduced by ~ 40% with NPs versus ~ 30% with free drug. By 48 h, this difference increased to ~ 50% vs. ~40%. At 72 h, the nanoformulation caused a 72.10 ± 4.30% reduction in *HSP90* mRNA, while free 17-AAG reduced it by only 48.30 ± 3.70% (*P* < 0.001). This progressive and sustained downregulation suggests that the nanoformulation improves intracellular drug availability through pH-triggered release and sustained retention, leading to more effective inhibition of *HSP90*. While this profound downregulation may suggest feedback mechanisms on HSF1, we emphasize that protein-level confirmation (e.g., Western blot) was not performed due to equipment constraints. Therefore, we limit our interpretation to the observed mRNA suppression.

The magnitude of *HSP90* mRNA downregulation (72.10% at 72 h) exceeds typical feedback responses reported for 17-AAG monotherapy, suggesting that our nanoformulation enhances pharmacodynamic effects through sustained intracellular drug delivery. While canonical *HSF1*-mediated feedback likely contributes, the progressive and time-dependent nature of suppression (40% → 72% over 72 h) implies prolonged target engagement due to pH-triggered release in acidic endo/lysosomal compartments. Although protein-level confirmation (e.g., Western blot for HSP90 or HSF1 phosphorylation) was not feasible due to technical constraints, the functional outcomes, including G2/M arrest, apoptosis, and synergy with trastuzumab, strongly support effective *HSP90* pathway inhibition at the transcriptional level. Melting curve analysis showed single sharp peaks for both β-actin (Tm = 83.90 °C) and *HSP90* (Tm = 78.20 °C), confirming the specificity of amplification and absence of primer-dimers (Fig. 7B).

qRT-PCR analysis showed a significant 72.10 ± 5.30% downregulation of HSP90 mRNA after 72 h (mean ± SEM, *n* = 3). Melting curve analysis confirmed amplification specificity (Fig. 7B).

### Synergistic effect with trastuzumab

**Fig. 8:**
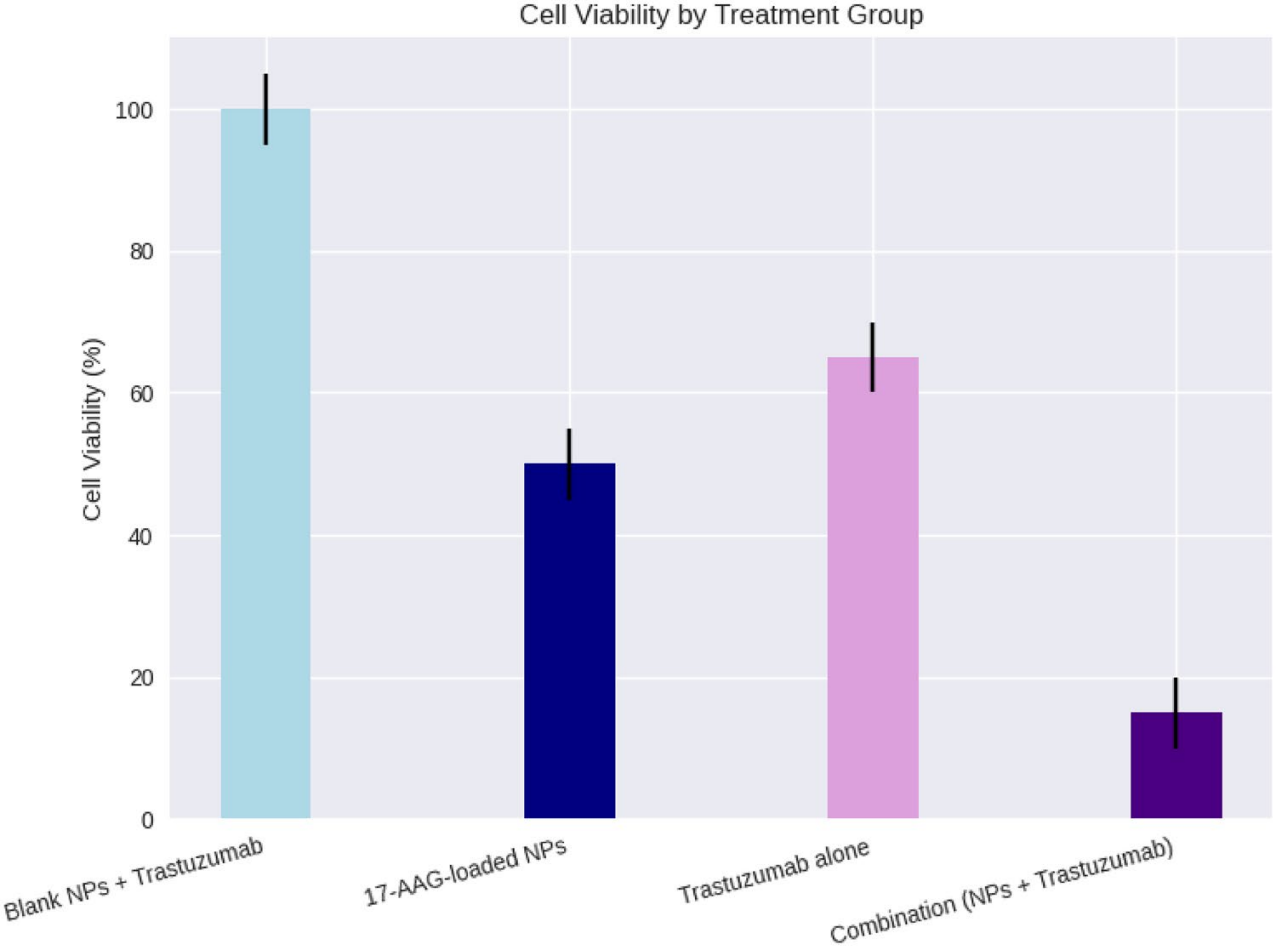
Synergistic cytotoxic effect of 17-AAG-loaded PEG-CS nanoparticles and trastuzumab on T47D cells. Cell viability was assessed by MTT assay after 72 h of treatment. Data are presented as mean ± SD (*n* = 3). The combination group (17-AAG-loaded NPs + trastuzumab) showed significantly reduced cell viability (15%) compared to treatment with 17-AAG-loaded NPs alone (50%), trastuzumab alone (35%), or blank NPs + trastuzumab (100%). Statistical significance: ****p < 0.0001 vs. Blank NPs + Trastuzumab group (one-way ANOVA with Tukey’s post-hoc test). The Combination Index (CI) calculated using CompuSyn software was 0.72, indicating strong synergy.

To evaluate the potential for combination therapy and overcome the limitations of monotherapy in HER2-positive breast cancer, T47D cells were treated with 17-AAG-loaded PEG-CS NPs, trastuzumab (10 µg/mL), or their combination. As shown in Fig. 8, the combination therapy resulted in a dramatic reduction in cell viability (85%) compared to either agent alone (NPs: ~50%, trastuzumab: ~65%), indicating a profound synergistic interaction.

The combination index (CI) was calculated using CompuSyn software based on the Chou-Talalay method^16^. At the IC₅₀ concentration, the CI was 0.72, which is well below the threshold of 1.00, confirming strong synergy between the nanoformulation and trastuzumab. This result is particularly significant as it demonstrates that the enhanced intracellular delivery of 17-AAG via PEGylated chitosan nanoparticles not only improves the efficacy of the drug itself but also potentiates its interaction with a clinically established monoclonal antibody.

This synergistic effect arises from a dual-targeting mechanism:

- **Trastuzumab** blocks HER2 signaling at the cell membrane level, inhibiting downstream survival pathways such as PI3K/AKT and RAS/MAPK.
- **17-AAG-loaded PEG-CS NPs** promote disruption of HER2 signaling via *HSP90* inhibition, leading to functional downregulation of HER2 and other oncogenic client proteins (e.g., AKT, RAF-1, CDK4).

By simultaneously targeting HER2 at both the extracellular and intracellular levels, this combination disrupts compensatory survival mechanisms and significantly enhances tumor cell killing. This approach is especially promising for overcoming acquired resistance to trastuzumab, a major clinical challenge in HER2^+^ breast cancer management.

Furthermore, the use of a nanocarrier system allows for lower effective doses of 17-AAG, potentially reducing systemic toxicity while maintaining or even enhancing therapeutic synergy. These findings highlight the translational potential of this nanoplatform as part of a rational, mechanism-driven combination therapy for refractory HER2-positive breast cancer.

## Discussion

Our study presents a pH-responsive PEGylated chitosan nanocarrier for the targeted delivery of 17-AAG, a potent *HSP90* inhibitor. Our choice of *HSP90* as a therapeutic target is further validated by recent network pharmacology analyses identifying *HSP90AA1* as a core hub target in breast and cervical cancers^11^. This system combines biocompatibility, pH-responsive release, and synergistic effects with trastuzumab, offering a rational strategy to improve the therapeutic profile of 17-AAG in breast cancer therapy.

The core design leverages chitosan as a natural, biodegradable polymer with mucoadhesive properties and inherent anti-tumor activity^5,6^. For instance, Wang et al. successfully formulated chitosan-based nanoparticles for paclitaxel delivery, demonstrating enhanced cellular uptake and cytotoxicity in breast cancer cells^17^.

The high encapsulation efficiency (86.40%) of 17-AAG in our PEG-CS NPs is another critical advantage. This is significantly higher than many previously reported systems. More recently, Zhou et al. developed pH-responsive polymeric micelles for co-delivery of 17-AAG and siRNA in HER2-positive breast cancer, achieving enhanced tumor accumulation and synergistic cytotoxicity under acidic conditions^18^. For example, Saxena et al. (2012) developed PLGA-PEG-folate nanoparticles for 17-AAG delivery and reported an encapsulation efficiency of only 8.25%^19^. Similarly, Gandomkar Ghalhar et al. (2014) used β-cyclodextrin complexes to improve 17-AAG solubility and showed enhanced HSP90 suppression in breast cancer cells^20^. However, their system lacked a nanostructured carrier for sustained release and tumor targeting, which our platform provides.

Modification with PEG confers ‘stealth’ properties^20^ while maintaining the cationic nature of chitosan, which facilitates interaction with the negatively charged membranes of cancer cells^21^. However, challenges such as batch-to-batch variability in chitosan properties and potential immunogenicity of PEGylated systems must be acknowledged.

The resulting nanoparticles exhibited a uniform spherical morphology with an average size of ~ 152 nm and a zeta potential of + 28.60 mV, which is optimal for both colloidal stability and cellular interaction. The size falls within the ideal range (100–200 nm) for passive tumor targeting via the enhanced permeability and retention (EPR) effect^8^ The positive charge further enhances tumor cell interaction, as demonstrated by the significantly higher cytotoxicity of our nanoformulation compared to free 17-AAG (IC_50_ = 2.30 µM vs. 6.90 µM after 72 h).

One of the most significant advantages of our system is its pH-responsive drug release profile. In vitro release studies showed that only 41.20% of 17-AAG was released at physiological pH (7.40) over 72 h, indicating minimal premature leakage during systemic circulation. In contrast, at acidic pH (5.50), mimicking the tumor microenvironment and endo/lysosomal compartments, 87.30% of the drug was released. This behavior is attributed to the protonation of chitosan’s amine groups at low pH, leading to polymer swelling, weakening of electrostatic interactions with TPP, and accelerated diffusion of the encapsulated drug^22^. This tumor-selective release mechanism not only maximizes drug concentration at the target site but also minimizes off-target toxicity, a major limitation of free 17-AAG.

Our nanoformulation demonstrated significantly enhanced cytotoxicity against T47D (HER2^+^) cells. This dramatic enhancement can be attributed to multiple factors promoting improved intracellular drug availability: (1) the cationic nature of chitosan, which facilitates interaction with the negatively charged cell membrane^20^, (2) sustained intracellular release of 17-AAG in acidic compartments, and (3) protection of the drug from degradation during circulation.

Furthermore, our system induced robust apoptosis (52.20% apoptotic population vs. 16.50% in controls) and caused G2/M cell cycle arrest (26.50% vs. 19.30%), both hallmarks of effective HSP90 inhibition. HSP90 is a master regulator of oncogenic client proteins, including HER2, AKT, CDK4, and survivin. Its inhibition triggers ubiquitin-mediated degradation of these clients, disrupting multiple pro-survival and proliferative signaling pathways^3–5^. The observed apoptosis and G2/M arrest are thus consistent with effective HSP90 suppression and downregulation of key cell cycle regulators such as CDK4 and cyclin B1.

Most importantly, qRT-PCR analysis revealed a 72.10% ± 5.30% downregulation of *HSP90* mRNA after 72 h of treatment with the nanoformulation, confirming transcriptional inhibition consistent with 17-AAG activity. While this profound downregulation may suggest feedback mechanisms on HSF1, we emphasize that protein-level confirmation (e.g., Western blot) was not performed due to equipment constraints. Therefore, we limit our interpretation to the observed mRNA suppression. The robust induction of apoptosis (52.2%) and G2/M arrest (26.5%) strongly supports caspase-dependent cell death initiated by HSP90 inhibition. HSP90 stabilizes key anti-apoptotic and cell cycle regulators (HER2, AKT, CDK1, survivin); their functional downregulation, even at the transcriptional level, disrupts pro-survival signaling and activates intrinsic apoptotic pathways, consistent with the known mechanism of 17-AAG.

One of the most compelling findings of this study is the synergistic effect (CI = 0.72) between our 17-AAG-loaded PEG-CS NPs and trastuzumab in T47D cells. Although T47D is not a canonical HER2-overexpressing line, the observed synergy provides strong functional evidence of HER2 pathway dependency in our subline consistent with prior in vivo evidence showing trastuzumab sensitivity in certain T47D-derived models^11^.

Trastuzumab, while effective, often leads to resistance due to persistent downstream signaling or incomplete receptor inhibition. In contrast, *HSP90* inhibition leads to the ubiquitin-mediated degradation of HER2 and multiple co-dependent oncoproteins, effectively dismantling the entire oncogenic network. Our nanoparticle system enhances this effect by ensuring sustained intracellular delivery of 17-AAG, resulting in prolonged suppression of *HSP90* function and greater depletion of client proteins. This dual-targeting strategy, membrane blockade by trastuzumab combined with intracellular disruption of HER2 signaling by 17-AAG-loaded NPs, represents a potential approach to help overcome trastuzumab resistance and improve therapeutic outcomes in HER2-expressing T47D cells. It also aligns with recent clinical evidence showing that combining *HSP90* inhibitors with trastuzumab is feasible and active in trastuzumab-refractory patients^7,8^. However, those trials used the free drug (tanespimycin), which suffers from poor pharmacokinetics and toxicity. Our nanoplatform offers a safer, more effective alternative by improving drug solubility, tumor targeting, and intracellular delivery. Recent advances in nanomedicine highlight the importance of stimuli-responsive systems for tumor-selective activation. As comprehensively reviewed by Yin et al., nanocarriers engineered to respond to tumor-specific stimuli (e.g., low pH, elevated enzymes, or redox imbalance) can significantly enhance therapeutic precision while minimizing off-target effects^9^. For instance, Gao et al. (2024) engineered polymeric nanoparticles that exploit tumor acidosis for on-demand drug release, while Chen et al. (2025) developed pH-activated micelles for co-delivery of doxorubicin and siRNA in HER2^+^ models both demonstrating that microenvironmental triggering is a robust strategy for precision therapy^2324^. Our PEG-chitosan system aligns with this paradigm, utilizing protonation-induced swelling at pH 5.5 to achieve selective 17-AAG release.

Recent work by Mahoutforoush et al. (2025) demonstrated that UiO-66 metal–organic frameworks functionalized with dermatan sulfate (a sulfated polysaccharide) enable dual active/passive targeting of cancer cells, combining receptor-mediated uptake (via CD44) with pH-responsive drug release in the acidic tumor microenvironment^25^. In this system, the high surface area and porosity of UiO-66 facilitate efficient drug loadingwhile the acidic pH of the tumor triggers framework degradation and controlled release of methotrexate.

Similarly, Chen et al. (2025) engineered citric acid/graphene oxide (CA/GO) nanocomposite beads that undergo acid-triggered swelling, leading to enhanced cellular internalization and potent anti-breast cancer activity^24^. The protonation of carboxyl groups in citric acid at low pH induces electrostatic repulsion and hydrophilic expansion, resulting in rapid drug release specifically within the tumor milieu.

These emerging platforms exemplify a unifying paradigm in modern nanomedicine: the integration of tumor microenvironment-responsive release with biomolecule-mediated active targeting to achieve spatial and temporal precision in drug delivery. Our PEGylated chitosan nanocarrier aligns with this paradigm by leveraging protonation-induced swelling for pH-selective 17-AAG release, while its cationic surface promotes non-specific electrostatic adhesion to negatively charged cancer membranes a form of passive targeting that complements receptor-based strategies. Moreover, unlike MOF or GO-based systems, our chitosan-based platform offers superior biodegradability, low cytotoxicity, and ease of functionalization, making it highly suitable for clinical translation. Critically, our system goes beyond passive or active targeting alone by demonstrating strong functional synergy with trastuzumab, offering a combinatorial approach to overcome resistance in HER2^+^ breast cancer. Beyond polymeric platforms, pH-responsive nanocomposite systems such as citric acid/graphene oxide (CA/GO) beads have demonstrated acid-triggered swelling and enhanced drug release specifically in the acidic tumor microenvironment, leading to improved cytotoxicity against breast cancer cells^24^. Similarly, redox-responsive nanoassemblies, such as disulfide bond-driven prodrug carriers developed by Zhang et al. (2025), highlight the growing trend of multi-stimuli-responsive platforms that combine pH, redox, or enzyme triggers to maximize tumor selectivity^16^. Gao et al. recently demonstrated that polymeric nanoparticles can achieve tumor-selective activation through microenvironmental triggers such as pH and redox gradients, enhancing therapeutic specificity while minimizing off-target effects^23^. Other redox-responsive strategies (such as disulfide bond-driven nanoassemblies of lipophilic epirubicin prodrugs) have demonstrated selective intracellular drug release in breast cancer models^16^. Our PEGylated chitosan nanocarrier aligns with this concept by exhibiting pH-responsive swelling and accelerated drug release under acidic conditions (pH 5.50), which closely resembles the endosomal/lysosomal environment of cancer cells.

Notably, our system achieves tumor selectivity without requiring HER2 overexpression, as evidenced by the wide therapeutic window between HER2-low T47D cells and HER2-low NHDFs. This underscores that the primary targeting mechanism is microenvironmental (pH/EPR), not receptor-mediated.

Furthermore, network pharmacology and experimental studies have underscored the pivotal role of HSP90 in oncogenic signaling pathways in breast cancer. HSP90 stabilizes key oncoproteins such as HER2, AKT, and RAF-1, and its inhibition leads to their ubiquitin-mediated degradation, disrupting proliferation, survival, and metastasis^26^. Our findings align with prior in vivo evidence from Solit et al. (2006), who demonstrated that 17-AAG induces tumor regression and apoptosis in HER2-overexpressing xenografts, particularly when combined with trastuzumab^11^.

Collectively, these findings position our PEGylated chitosan nanoplatform within a growing trend of smart, multifunctional nanosystems designed for precision oncology. By integrating pH-responsiveness, high drug loading, and synergistic potential with targeted therapies like trastuzumab, our system offers a rational and translational strategy for overcoming the limitations of conventional chemotherapy in refractory HER2^+^ breast cancer.

It should be noted that mycoplasma contamination testing was not performed due to technical limitations. However, all cell cultures were maintained under sterile conditions, showed normal morphology and growth kinetics throughout the experiments, and were used within low passage numbers (8–12 for NHDFs, and < 20 for T47D), which reduces the likelihood of contamination. This study has some limitations. First, due to equipment constraints, Western blot analysis was not performed to confirm protein-level changes in HER2 and HSP90. Second, cytotoxicity was assessed in normal human dermal fibroblasts (NHDFs) rather than mammary epithelial cells (e.g., MCF-10 A), which would better model HER2 expression in healthy breast tissue. However, NHDFs are a widely accepted normal control in nanotoxicity studies^20,22^, and the > 20-fold therapeutic window strongly supports tumor-selective delivery via EPR and pH-responsiveness not HER2 overexpression alone. Future work will focus on validating these results in animal models and assessing long-term safety.

A comparative summary of key parameters across reported 17-AAG nanocarrier systems, including our PEGylated chitosan platform, is provided in Table 1.

**Table 1:** A direct comparison with other 17-AAG nanocarriers highlights the superior performance of our system.

Nanocarrier system	Particle size (nm)	Encapsulation efficiency (EE%)	pH-responsive release	IC₅₀ in breast cancer cells (µM)	Targeting strategy	Synergy with trastuzumab?
PLGA-PEG micelles [12]	85–110	78%	Yes	4.50	Passive (EPR)	No
PLGA nanoparticles [20]	160–200	65–75%	Yes	6.20	Passive (EPR)	Not reported
Citric acid/graphene oxide (CA/GO) beads [22]	~ 200 (bead size)	Not reported	Yes (acid-triggered swelling)	~ 3.00	Passive (EPR) + pH-responsive uptake	Not reported
Dermatan sulfate-UiO-66 MOFs [25]	120–150	~ 80%	Yes (pH-triggered degradation)	~ 4.00	Active (CD44-mediated) + Passive + pH-responsive	Not reported
**PEGylated chitosan NPs (this study)**	**152 ± 14**	**86.40 ± 3.20%**	**Yes (87.30% at pH 5.40 vs. 41.20% at pH 7.40)**	**2.30 (in T47D**,** 72 h)**	**Passive (EPR) + pH-responsive**	**Yes (CI = 0.72)**

bold values indicate superior performance or key advantages of our nanocarrier system compared to other reportedsystems.

## Conclusion

This study presents a pH-responsive PEGylated chitosan nanoplatform for targeted delivery of 17-AAG in breast cancer. The system improved solubility, enables pH-responsive tumor-selective release, and enhances cytotoxicity, apoptosis induction, cell cycle arrest, and HSP90 suppression, indicative of effective intracellular delivery. The observed synergy with trastuzumab offers a strategy with therapeutic potential to overcome resistance in HER2^+^ disease. The biocompatibility and modular design support future functionalization (e.g., antibody conjugation) and in vivo translation. While only in vitro studies were performed, this work lays the foundation for developing safer, more effective nanotherapies for refractory breast cancers.

## Supplementary Information

Below is the link to the electronic supplementary material.

Supplementary Material 1

## Data Availability

The data that support the findings of this study are available from the corresponding author upon reasonable request.
