# Supplementary material for: Development of a pH-responsive pegylated chitosan nanocarrier for targeted delivery of 17-AAG and synergistic therapy in HER2+ breast cancer
Source: Sci Rep. 2025 Dec 2;16:909. doi: 10.1038/s41598-025-30507-2 (PMC12783725; doi:10.1038/s41598-025-30507-2)
Supplement: Supplementary file 1 — Supplementary Material 1 [file 41598_2025_30507_MOESM1_ESM.docx]

**Figure S1**

**
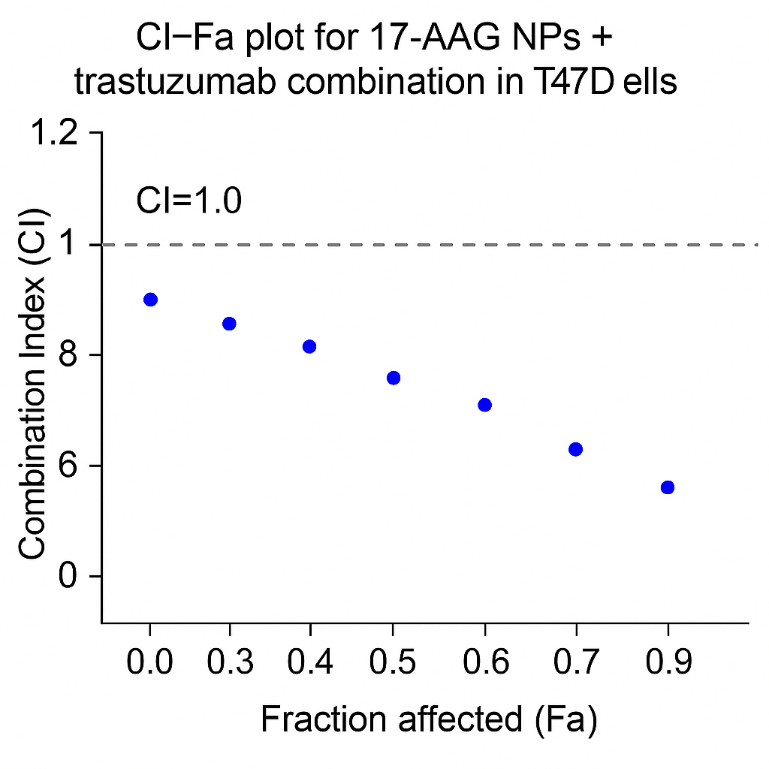
**

**Figure S1. CI–Fa plot for the combination of 17-AAG-loaded PEG-CS NPs and trastuzumab in T47D cells.**

The Combination Index (CI) is plotted against the fractional effect (Fa). All data points fall below the line CI = 1.0, indicating strong synergy across the entire effect range (Fa = 0.1 to 0.9).
